# Impairment in Theory of Mind in Parkinson's Disease Is Explained by Deficits in Inhibition

**DOI:** 10.1155/2019/5480913

**Published:** 2019-05-28

**Authors:** Jennifer A. Foley, Claire Lancaster, Elena Poznyak, Olga Borejko, Elaine Niven, Thomas Foltynie, Sharon Abrahams, Lisa Cipolotti

**Affiliations:** ^1^National Hospital for Neurology and Neurosurgery, Queen Square, London, UK; ^2^UCL Institute of Neurology, Queen Square, London, UK; ^3^Psychology, University of Dundee, Dundee, UK; ^4^Department of Clinical and Movement Neurosciences, UCL Institute of Neurology, Queen Square, London, UK; ^5^Human Cognitive Neuroscience–PPLS, University of Edinburgh, Edinburgh, UK; ^6^Centre for Cognitive Ageing and Cognitive Epidemiology, University of Edinburgh, Edinburgh, UK; ^7^Anne Rowling Regenerative Neurology Clinic, University of Edinburgh, Edinburgh, UK; ^8^Dipartimento di Psicologia, University of Palermo, Palermo, Italy

## Abstract

**Objective:**

Several studies have reported that people with Parkinson's disease (PD) perform poorly on tests of ‘Theory of Mind' (ToM), suggesting impairment in the ability to understand and infer other people's thoughts and feelings. However, few studies have sought to separate the processes involved in social reasoning from those involved in managing the inhibitory demands on these tests. In this study, we investigated the contribution of inhibition to ToM performance in PD.

**Methods:**

18 PD patients and 22 age-matched healthy controls performed a ToM test that separates the ability to infer someone else's perspective from the ability to inhibit one's own. Participants also completed a battery of standard measures of social and executive functioning, including measures of inhibition.

**Results:**

The PD patients performed worse on the ToM test only when the inhibitory demands were high. When the level of inhibition required was reduced, there were no significant group differences. Furthermore, executive impairments in PD patients were limited to measures of inhibition, with disadvantages associated with poorer ToM performance in this group.

**Conclusions:**

This study provides convincing evidence that the apparent impairment observed on ToM tests in PD is explained by deficits in inhibition.

## 1. Introduction

Several studies have reported that people with Parkinson's disease (PD) perform poorly on tests assessing the ability to infer the beliefs, desires, and intentions of others [1–3]. These functions fall under the umbrella term of “Theory of Mind” (ToM), considered essential for the development and maintenance of successful social relationships [4].

ToM has been separated into cognitive and affective components [5]. Cognitive ToM is the ability to identify others' beliefs and intentions, and affective ToM is the ability to empathise with others' emotional states. In PD, there are reports of impairments in both [1, 2], but these have been inconsistent [6–8], with some reporting impairment only in cognitive ToM [7–11] or advanced disease [8]. The variation in results appears to depend upon the specific measure used and severity of PD in the cohort tested [12]. This would suggest that the incidental processing demands of the individual tests may be contributing to the observed variation.

This variation may be, at least in part, explained by the varying demands that each test places upon executive function. Although the precise role of executive function in performance on ToM tests remains debated, some have argued that ToM is simply the reflection of “domain-general” executive functions within a social realm [13]. Alternative accounts have construed ToM as a specialised process, involving dedicated or “domain-specific” ToM computations, distinct from executive functions [14–16]. Others still have suggested that performance on ToM tests involves both domain-general executive functions and domain-specific ToM processes [17].

There are reports of a double dissociation between ToM and executive functions: impaired ToM with preserved executive functions [14, 18, 19] and preserved ToM with impaired executive functions [20, 21], suggesting separability of function. However, this apparent independence may betray an insufficiently broad assessment of the range of functions underpinned by executive control [22]. In particular, there is mounting evidence to suggest that the executive function of inhibition is crucial for performance on ToM tests. Several studies have found inhibition to be highly correlated with and predictive of performance on ToM tests in children [23–25] and adults [26]. In keeping with this, closer inspection of the aforementioned reports of preserved ToM with impaired executive functions reveals that this occurred in the presence of intact performance the Stroop Colour Word Test of inhibition [20] or in the absence of any measure of inhibition [21].

Samson and colleagues [27, 28] argue that standard tests of ToM lack the capacity to identify the specific cognitive function underlying impaired performance. For example, a canonical test of ToM is the “false belief” test. During this test, participants are asked to listen to a story in which a character hides an object and then leaves the room. When this character is outside of the room, a second character moves the hidden object to a new location. Participants are then asked where the first character will think the object is. Samson and colleagues argue that in order to answer this question correctly, participants must first inhibit their own knowledge of where the object is (self-perspective) in order to focus on the first character's false belief (other-perspective). This necessitates high demands upon attentional and inhibitory functions.

The neuropsychological profile of PD is characterised by deficits in attentional and executive function, most notably in inhibition [29–33]. Speed of processing is also reduced [34–36]. As PD progresses, other cognitive domains become increasing affected, with additional impairments in memory and visual processing [37, 38]. Thus, it remains unclear how much of the apparent impairment on ToM tests in PD may be explained by deficits in more general cognitive abilities, and particularly the executive function of inhibition.

The aim of the current study is to assess ToM abilities in PD when controlling for incidental inhibitory demands. In order to do this, we designed a false belief test based upon that described by Samson and colleagues [27, 28, 39], which directly manipulates the level of inhibition involved. In addition, we also assessed participants on standard ToM and cognitive tests, including several measures of executive functioning, including inhibition, and measures of mood, in order to investigate the relationship between these and performance on the experimental measure.

## 2. Methods

### 2.1. Participants

A total of 18 patients with idiopathic PD and 22 healthy age-matched controls took part in this study. All patients were recruited from the National Hospital for Neurology and Neurosurgery, Queen Square, London. All fulfilled Queen Square Brain Bank criteria for PD and had no diagnosis of dementia. All patients were receiving dopaminergic medication and tested under their usual medication conditions. The healthy controls were recruited amongst patients' spouses or relatives or through local advertisement. No participant had significant neurological or psychiatric history. The characteristics of the two groups are shown in Table 1.

The research was done in accordance with the Helsinki declaration and the Institute of Neurology Joint Research Ethics Committee UCLH, NHS Trust Research and Development Directorate.

### 2.2. Procedure

All of the patients and healthy controls completed the following assessments.

#### 2.2.1. ToM Test: High and Low Inhibition Conditions

This test was adapted from Samson and colleagues [27, 28, 39]. In our version, participants completed 12 trials in each condition (high and low inhibition), presented in a pseudorandomized order. The high inhibition condition is similar to a classical false belief test. Here, the participant is shown a woman seeing an object placed inside one of three identical boxes. She leaves the room and in her absence, the location of the boxes is swapped. The woman then returns, and the participant is asked where she will look for the object. In order to answer correctly, the participant must not only infer the woman's false belief but also inhibit their own perspective of knowing the true location of the object.

In the low inhibition condition, the participant sees the woman looking inside the three boxes but is not shown which of the boxes contains the object. The woman then leaves the room, and as before, the location of the boxes is swapped. The woman then returns and offers the participant a clue about the location of the object by pointing to one of the boxes. The participant is then asked where she will look for the object. In order to answer correctly, the participant must infer the woman's false belief to choose the box she has selected. Crucially, in this condition, the participant does not have to inhibit knowledge of the object's true location. Thus, for each trial, the participant is asked where the woman will look for the object, assessing the participant's ability to infer a false belief. The participant is also asked where is the true location of the object, as a control measure to assess comprehension.

We modified the original test in two ways. Firstly, the original videos used only young actors. As there is evidence of an ”own-age bias” in face processing [40–42], the videos were re-shot to include older actors. Secondly, as the original test involved choosing between only two boxes, it invoked a binary response choice and required a great number of trials per condition to reduce the influence of chance, with an administration time of at least two hours. In the present study, the number of boxes was increased to three, allowing a reduction in the number of trials and administration time. When the correct answer could have been one of two possible locations, a response indicating either or both locations was accepted. Each question therefore had a maximum possible score of 12.

#### 2.2.2. Standard Measures of ToM and Social Cognition

ToM was assessed using the Reading the Mind in the Eyes Test, Revised Version (RMET; [43]). On this test, participants were shown the eye regions of actors and asked to identify their mental state from one of the four possible responses. Social cognition was also assessed using the Ekman 60 Faces [44]. Participants were shown the faces of 10 actors and asked to identify the emotion expressed from one of six possible responses: happiness, sadness, disgust, fear, surprise, and anger.

#### 2.2.3. Executive Functioning

Executive functioning was assessed using measures of attentional flexibility, updating of information in working memory, and inhibition of pre-potent responses respectively. Attentional flexibility, or set-shifting, was assessed using the Plus/Minus test [45] and the Brixton Spatial Anticipation Test [46]. Updating of information in working memory was assessed using the Digit Span subtest from the Wechsler Adult Intelligence Scale, Third Edition (WAIS-III; [47]). Inhibition of prepotent responses was assessed using the Stroop Colour Word Test [48], the Hayling Sentence Completion Test [46], and the Elevator Counting with Distraction subtest from the Test of Everyday Attention [49]. In addition, measures of phonemic (FAS; [50]) and semantic (animals; [50]) verbal fluency were also used.

#### 2.2.4. Background Cognitive Tests

Other cognitive tests administered included the Story subtests from the Adult Memory and Information Processing Battery [51] and the Symbol Digit Modalities Test [52].

#### 2.2.5. Mood

Mood state was assessed using the Hospital Anxiety and Depression Scale (HADS; [53]) and Apathy Evaluation Scale (AES; [54]).

### 2.3. Statistical Analysis

Mean and standard deviations were calculated for each of the variables. Normality of distribution was assessed using the Kolmogorov–Smirnov test and, if significant, by examining the *z*-scores for skewness and kurtosis. Homogeneity of variance was assessed using Levene's test. Unless otherwise stated, all data met the assumptions of normality and homogeneity of variance. It was not possible to conduct a mixed analysis of variance because of insufficient homogeneity of variance despite square root transformation. Therefore, scores were compared between groups using *t*-tests for related samples and independent *t*-tests, or Wilcoxon signed-ranks and Mann–Whitney analyses, as appropriate, corrected for multiple comparisons. Scores were also analysed using Pearson's correlational, principal components, and multiple regression analyses to explore the relationships between performance on measures of ToM and executive functioning, corrected for multiple comparisons where appropriate. All tests were conducted using IBM SPSS Statistics Data Editor version 24.

## 3. Results

### 3.1. Participants

The two groups were matched in age (*t* (28.38) = −0.01, *p*=0.09), gender (*χ*^2^ (1) = 0.85, *p*=0.27), and NART Predicted Full Scale IQ (*t* (37) = 0.77, *p*=0.45).

### 3.2. ToM Test: High and Low Inhibition Conditions

Mean performance on the ToM test in the two groups is shown in Table 2.

#### 3.2.1. False Belief Test

**Figure 1:**
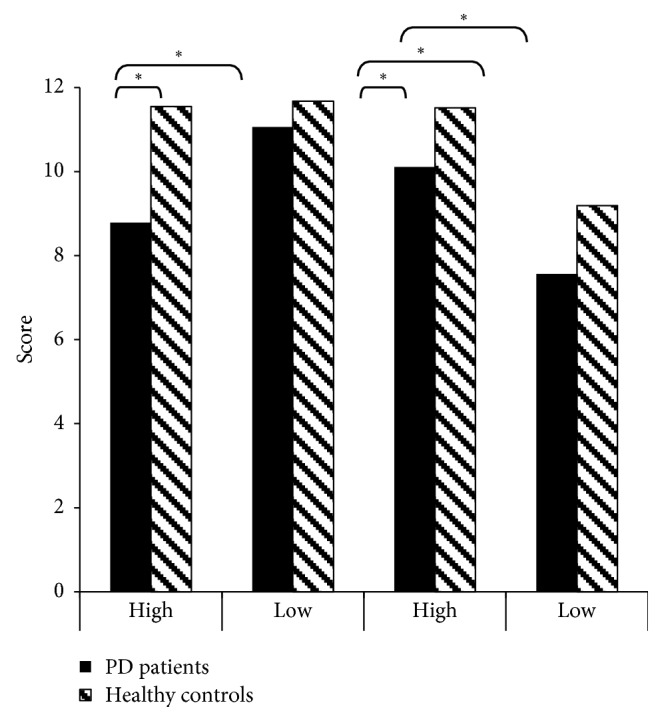
Performance on the ToM test in high and low inhibition conditions in the two groups.

As shown in Figure 1, Wilcoxon signed-rank tests revealed that the PD patients performed worse in the high inhibition condition than in the low inhibition condition (*Z* = −2.40, *p* < 0.05). There was no such difference in the healthy controls (*Z* = −0.91, *p*=0.37).

Mann–Whitney tests revealed that the PD patients performed significantly worse than the healthy controls on the false belief test in the high inhibition condition (*U* = 57.00, *p* < 0.001). There were no significant group differences in the low inhibition condition (*U* = 153.00, *p*=0.60).

#### 3.2.2. True Location Test

Wilcoxon signed-rank tests also revealed that both groups performed worse on the true location test in the low inhibition condition than in the high inhibition condition (age-matched: *Z* = −3.13, *p* < 0.01; PD:*Z* = −2.74, *p* < 0.01).

Mann–Whitney tests revealed that the PD patients performed significantly worse than the healthy controls on the true location test in the high inhibition condition (*U* = 88.50, *p* < 0.01). There were no significant group differences in the low inhibition condition (*U* = 124.00, *p*=0.69).

### 3.3. Standard Measures of ToM, Social Cognition, and Executive Function

Mean scores on the standard measures of ToM, social cognition, and executive function are reported in Table 3.

Independent *t*-tests revealed no significant group differences in performance on the Ekman (*t* (36) = −0.96, *p*=0.34). However, PD patients performed significantly worse than the healthy controls on the RMET test (*t* (37) =3.15, *p* < 0.01). The PD group also performed significantly worse on one measure of executive functioning only, namely, the Hayling (*t* (27.63) = 14.13, *p* < 0.01).

### 3.4. Background Cognitive Tests and Mood

Scores on the background cognitive tests and measures of mood are reported in Table 4.

Independent *t*-tests revealed that the PD patients performed significantly worse than healthy controls on both AMIPB immediate (*t* (35) = 3.85, *p* < 0.001) and delayed story recall (*t* (32.64) = 3.08, *p* < 0.01). There were no other significant group differences. There were no significant group differences in mood scores.

### 3.5. Relationship between ToM and PD Disease Characteristics

Pearson correlations were conducted to investigate the relationship between the PD patients' performance on the ToM test and their PD disease characteristics (dopamine dosage and disease duration). This revealed that higher dopamine dosages were associated with poorer performance in the high inhibition condition of the false belief test (*r* = −0.50, *p* < 0.05). There were no other significant associations.

### 3.6. Relationship between ToM and Executive Functioning

A principal components analysis with varimax rotation was also conducted to determine the relationship between the performance on the false belief test and measures of executive function. As shown in Table 5, this analysis extracted four independent factors. The first factor comprised scores on tests tapping inhibitory functions, namely, the false belief test in the high inhibition condition, the Stroop, Hayling, and Elevator Counting with Distraction tests, as well as the Brixton and both measures of verbal fluency to a lesser extent. The second factor reflected primarily working memory, with performance on the False Belief test in the low inhibition condition, Digit Span, and the Brixton and FAS fluency to lesser extents. The third factor only involved the set-shifting test of Plus/Minus ratio, whereas the fourth factor reflected its associations with the False Belief test in the low inhibition condition and verbal fluency.

Pearson correlations were also conducted to explore further the relationship between ToM and executive functioning. These revealed that performance on the false belief test in high inhibition condition correlated with performance on the Hayling (*r* = 0.52, *p* < 0.01) and Elevator Counting with Distraction tests only (*r* = .61, *p* < 0.001). Performance in the low inhibition condition was not associated with performance on any measures of inhibition or executive function. There were also no significant correlations between performance on the object location control test in either condition and performance on any measure of executive functioning.

Multiple regression analysis revealed that performance on the Hayling and Elevator Counting with Distraction tests was a significant predictor of performance on the False Belief test in the high inhibition condition (*F*_2,27_ = 10.28, *p* < 0.001). Together, the two tests accounted for 39.0% of the variance.

## 4. Discussion

The current study found that patients with PD performed significantly worse than age-matched healthy controls on two measures of ToM: our ToM test and RMET. At first glance, this finding lends support to the suggestion that, in PD, there is an underlying deficit in the ability to infer the beliefs, desires, and intentions of other people, consistent with other studies [1, 2] [7–9, 11, 12]. However, the main aim of the present study was to determine how much of this apparent impairment may be explained by the incidental processing demands that ToM tests incur, most notably in inhibition. Full neuropsychological testing revealed that the PD patients demonstrated impairment in executive functions and specifically in inhibition. Although PD patients demonstrated lower scores on all tests of executive functions, performance was only significantly reduced on one test of inhibition: the Hayling. This supports previous findings that PD is characterised by deficits in executive function and particularly inhibition [29–33]. Strikingly, when we manipulated our ToM test to reduce the level of inhibition required, there were no longer any group differences in performance. Further analyses also revealed that performance on the high inhibition condition of the ToM test was negatively correlated with greater impairment on measures of inhibition and, indeed, performance on the ToM test was predicted by performance on measures of inhibition. Furthermore, factor analysis confirmed that performance on the high and low inhibition conditions dissociated, with performance on the high inhibition condition loading upon inhibition, whereas performance on the low inhibition condition loaded upon working memory.

These findings suggest that, in PD, there is no impairment in ToM per se but rather the executive functions that support performance on ToM tests are diminished: deficits in inhibition underlie the impairment in ToM. This finding may help explain the inconsistency observed on tests of social cognition in both the current and previous studies. For example, although patients performed poorly on the RMET and the experimental measure of ToM, there were no significant group differences in recognising facial emotional expressions on the Ekman. Emotion recognition is thought to be a close correlate of social cognition [55] and impaired performance which is a diagnostic marker of frontotemporal dementia [56]. The preservation of emotion recognition affirms our finding that deficits in social cognition are not constitutional to PD, but rather reflect the incidental demands of the tests used. One previous study that reported emotion recognition deficits found, rather counterintuitively, performance to be worse in those with less advanced PD, but who were unmedicated at time of testing [57]. Other studies have also shown little correlation between performance on this test and severity of motor symptoms [58]. This suggests that the proposed deficit in emotion recognition is not an inherent trait of PD, but rather a state-based epiphenomenon, with performance on such tests likely reflecting their incidental processing demands. Indeed, Bull et al. [59] found that performance on the RMET test was disrupted when participants were required to perform a secondary task involving inhibitory processing. This disruption was not witnessed when the secondary task involved other executive functions, namely, working memory or switching, nor when the task did not require attribution of mental states. Thus, this test's apparent reliance upon inhibitory processing may explain why our PD patients were impaired on this test.

The finding that there are only ToM deficits in PD when the ToM test places greater demand upon inhibition supports previous findings of a relationship between ToM performance and executive function [8, 12]. For example, Eddy et al. [60] also found that people with PD demonstrated less impairment on ToM tests when the executive load was reduced. Specifically, they found that performance on longer, but not shorter, verbal tests of ToM was associated with verbal working memory. In contrast with the current study, they argue that working memory and executive functioning deficits do not wholly explain ToM performance. It is important to note, however, that they omitted to include a measure of inhibition in this experiment. In an additional experiment, they did include measures of inhibition but failed to find any significant group differences in either inhibition or ToM, supporting the argument that performance on these is intrinsically linked in PD.

Anderson et al. [61] also reported the preservation of social cognition in PD, with poor performance tests of social cognition only occurring in the context of greater executive dysfunction. They propose that when faced with an everyday social problem, people with PD may have greater difficulty inhibiting any previously unsuccessful problem-solving strategies, resulting in the generation of fewer viable alternatives and reliance upon prepotent responding. Our study extends this finding to provide evidence that poor performance on tests of ToM tests in PD is explained by deficits in inhibition. The impact of executive load may also explain why the PD patients also performed worse on the control question within the ToM test. As the original [28] test was modified to include three rather than two boxes, the control question may have inadvertently become more challenging and involve greater processing demands.

It is important to note that overall cognitive load cannot account for the PD deficits in the high inhibition condition. The most challenging subtest on our ToM test appears to have been the true location test in the low inhibition condition, with both participant groups performing significantly worse on this subtest than in the high inhibition condition. Yet, no significant group differences were found here, supporting the suggestion that it is the deficits in inhibition that lead to the PD-specific impairments on the false belief task.

Our findings are in keeping with the known neuropathology of PD. Studies have repeatedly shown that PD is characterised by reductions in frontal lobe volume, metabolism, and connectivity [62–64]. Frontal areas are known to be critical for inhibition [65–67], with right frontal areas particularly involved in the inhibition of one's own perspective [28, 68]. The frontal lobe is also thought to be involved in ToM [69]. However, frontal lobe damage does not necessarily result in impairments in ToM [20] and inhibition is increasingly recognised as important for ToM [23, 24] and Doenyas et al. [26]. Therefore, we argue that our patients' deficits on our tests of ToM may be explained by their impairments in inhibition associated with the known frontal lobe damage in PD.

A limitation of our study is the small sample size. All previous research using this methodology has been limited to single cases or very small case series [27, 28, 39], and therefore, although our patient group size is modest, we hope it will provide a first step towards larger patient group studies.

Future research may wish to expand upon our methodology to include a further control condition that would not require the inference of a false belief, but rather assess the ability to follow the object being moved. This would provide a vigilance control condition enabling better isolation of the cognitive processes involved in the false belief task. However, increasing the number of conditions would necessitate a longer administration time and possibly increase testing fatigue.

In conclusion, this study shows that deficits observed on ToM tests in PD may be explained by deficits in inhibition. Poor performance on these tests therefore does not indicate impairment in social cognition, but more likely deficits in managing the complex processing demands that these tests involve.

## Figures and Tables

**Table 1:** Characteristics of PD patients and healthy controls.

	PD patients (*n* = 18)	Age-matched controls (*n* = 22)
Gender (male)	10, 55.6%	9, 40.9%
Age (years)	63.83 ± 10.73	63.81 ± 7.09
NART predicted IQ	117.94 ± 6.64	119.41 ± 5.33
Age of onset (years)	57.56 ± 10.70	—
Duration of illness (years)	6.11 ± 3.07	—
Dopamine dosage (mg)	655.15 ± 450.34	—

**Table 2:** Performance on the ToM test in the two groups (mean ± SD).

Test	Condition	PD patients (*n* = 18)	Healthy controls (*n* = 22)
False belief	High inhibition	8.78 ± 2.53	11.55 ± 0.69^*∗∗*^
	Low inhibition	11.06 ± 1.83	11.68 ± 0.58

True location	High inhibition	10.11 ± 1.64	11.52 ± 0.68^*∗*^
	Low inhibition	7.56 ± 2.94	9.19 2.34

^*∗*^ *p* < 0.01; ^*∗∗*^*p* < 0.001.

**Table 3:** Performance on the standard measures of ToM, social cognition, and executive functioning in the two groups (mean ± SD).

		PD patients (*n* = 18)	Healthy controls (*n* = 22)
Social	Ekman	49.42 ± 5.34	47.91 ± 4.30
Cognition	RMET	23.59 ± 3.28	26.77 ± 3.01^*∗*^
Inhibition	Stroop—total	85.83 ± 19.49	91.53 ± 17.83
	Hayling—total scaled score	16.35 ± 2.12	18.70 ± 1.22^*∗*^
	Elevator counting with distraction	7.65 ± 3.02	8.94 ± 1.21
Set-shifting	Plus/Minus ratio	1.38 ± 0.27	1.43 ± 0.17
	Brixton—overall score	4.44 ± 2.50	4.95 ± 2.54
Updating	Digit span: forwards and backwards	7.65 ± 3.02	8.94 ± 1.21
Fluency	FAS—total	40.00 ± 14.25	40.95 ± 10.80
	Animals—total	19.06 ± 4.25	21.60 ± 2.58

^*∗*^ *p* < 0.01; RMET: reading the mind in the eyes test, revised version.

**Table 4:** Performance on the background cognitive tests and mood assessments in the two groups (mean ± SD).

	PD patients (*n* = 18)	Healthy controls (*n* = 22)
AMIPB story immediate	27.94 ± 7.23	38.80 ± 9.51^*∗∗*^
AMIPB story delayed	24.00 ± 6.94	34.29 ± 12.22^*∗*^
AMIPB story retained	85.49 ± 9.76	90.41 ± 9.05
SDMT	40.12 ± 8.37	44.71 ± 5.82
HADS—anxiety	7.53 ± 3.64	5.19 ± 3.23
HADS—depression	4.00 ± 2.30	3.52 ± 2.80
Apathy	25.67 ± 3.37	24.50 ± 5.61

^*∗*^ *p* < 0.01; ^*∗∗*^*p* < 0.001. AMIPB: Adult Memory and Information Processing Battery; SDMT: Symbol Digit Modalities Test; HADS: Hospital Anxiety and Depression Scale.

**Table 5:** Principal component analysis (with varimax rotation) for underlying factors on tests of executive function and false belief.

	Factor 1	Factor 2	Factor 3	Factor 4
False belief—high inhibition	0.81			
False belief—low inhibition		0.67		0.52
Stroop—total	0.81			
Hayling—overall score	0.77			
Elevator counting with distraction	0.82			
Plus/minus ratio			0.80	0.44
Brixton—overall score	0.67	0.47		
Digit span: forwards—backwards		0.65		
FAS—total	0.45	−0.58		0.42
Animals—total	0.61			0.42

## Data Availability

The neuropsychological data used to support the findings of this study are restricted by the Institute of Neurology Joint Research Ethics Committee UCLH in order to protect patient privacy. Data are available from the corresponding author for researchers who meet the criteria for access to confidential data.
